# To Become or Not to Become Tumorigenic: Subventricular Zone *Versus* Hippocampal Neural Stem Cells

**DOI:** 10.3389/fonc.2020.602217

**Published:** 2020-11-27

**Authors:** Ángela Fontán-Lozano, Sara Morcuende, Mª América Davis-López de Carrizosa, Beatriz Benítez-Temiño, Rebeca Mejías, Esperanza R. Matarredona

**Affiliations:** Departamento de Fisiología, Facultad de Biología, Universidad de Sevilla, Seville, Spain

**Keywords:** neurogenesis, glioblastoma, neural stem cells, oncogenicity, cancer-driver mutations

## Abstract

Neural stem cells (NSCs) persist in the adult mammalian brain in two neurogenic regions: the subventricular zone lining the lateral ventricles and the dentate gyrus of the hippocampus. Compelling evidence suggests that NSCs of the subventricular zone could be the cell type of origin of glioblastoma, the most devastating brain tumor. Studies in glioblastoma patients revealed that NSCs of the tumor-free subventricular zone, harbor cancer-driver mutations that were found in the tumor cells but were not present in normal cortical tissue. Endogenous mutagenesis can also take place in hippocampal NSCs. However, to date, no conclusive studies have linked hippocampal mutations with glioblastoma development. In addition, glioblastoma cells often invade or are closely located to the subventricular zone, whereas they do not tend to infiltrate into the hippocampus. In this review we will analyze possible causes by which subventricular zone NSCs might be more susceptible to malignant transformation than their hippocampal counterparts. Cellular and molecular differences between the two neurogenic niches, as well as genotypic and phenotypic characteristics of their respective NSCs will be discussed regarding why the cell type originating glioblastoma brain tumors has been linked mainly to subventricular zone, but not to hippocampal NSCs.

## Introduction

Gliomas constitute the most common and lethal primary tumor in the central nervous system (CNS). The World Health Organization (WHO) classified CNS tumors by their histological origin, molecular parameters and malignancy (1, 2). Glioblastoma multiforme (GBM) is considered as the highest grade (Grade IV) astrocytoma, characterized by poorly differentiated cells with microvascular proliferation, pseudopalisading necrosis, abundant mitoses and pleomorphic cells. This type of glioma also shows a high degree of phenotypic, genomic and transcriptional heterogeneity (3, 4). Extremely invasive, GBMs cannot be completely resected by surgery, and are resistant to conventional therapies, including chemotherapy and radiation. As a consequence, the prognosis for GBM patients is very poor, with an average survival rate of about 14–15 months even after intensive treatment (5, 6).

The vast majority of GBMs (about 90% of cases) are primary GBMs that rapidly develop *de novo* in elderly patients without radiological or histological evidence of pre-existing less-malignant precursor lesion. About 10% of the cases correspond to secondary GBMs progressing from lower grade gliomas and preferentially arise in younger patients (2). Although both GBM types are histologically indistinguishable, secondary GBMs are unequivocally characterized by the presence of *IDH1* (isocitrate dehydrogenase) mutations (7). For this reason, primary and secondary GBMs can also be named as IDH-wild type and IDH-mutant GBM, respectively (2). Primary (IDH-wild type) GBMs typically present epidermal growth factor receptor (*EGFR*) amplification and loss of the tumor suppressor phosphatase and tensin homolog (*PTEN*). Inactivation of the tumor suppressors *TP53* (coding a protein called tumor protein 53 or p53) and *NF1* (neurofibromin 1), or mutations in the promoter of *TERT* (telomerase reverse transcriptase) are also commonly identified in both GBM types (3, 8, 9).

Identification of the cell of origin for GBM, this is, the cell type that acquires the initial tumorigenic mutation, is a fundamental issue for understanding the etiology of the disease and for developing early prognostic markers and preventive therapies. Specifically, the cell of origin in IDH-wild type GBM has been much more object of debate since, in contrast to IDH-mutant GBM, the wild type arises without any precursor disease. One of the hypotheses states that neural stem cells (NSCs) remaining in the adult brain could be the cell of origin of this devastating disease. NSCs are found in two neurogenic niches: the subventricular zone (SVZ), lining the walls of the lateral ventricles, and the subgranular zone (SGZ), in the dentate gyrus of the hippocampus (10). Recent evidence has shown that SVZ-derived NSCs might be the cell type harboring the cancer-driver mutations that lead to GBMs (11). In contrast, to date, no substantial data support the contribution of hippocampal-derived NSCs in the development of these malignant tumors. Remarkably, in mouse models of malignant gliomas and in GBM patients, the hippocampus appears to be a region spared from GBM invasion whereas the SVZ is a site for preferred infiltration of this type of tumor (12).

In this article we will analyze differences in these two neurogenic niches, as well as between the NSC population residing in each of them, which might explain why the cell of origin of IDH-wild type GBM has been linked mainly to the SVZ, but not to hippocampal NSCs.

## The Adult Brain Neurogenic Niches in Mammals: Subventricular Zone and Hippocampus

In the majority of species of terrestrial mammals, adult CNS new neurons can be generated from NSCs residing in two specific regions: the SVZ and the SGZ in the dentate gyrus of the hippocampus (10, 13). Young neurons produced in the SVZ migrate over an extended distance along the rostral migratory stream toward the olfactory bulb, where their final differentiation takes place (14). In contrast, neuroblasts generated in the SGZ mature into granule cells within the same hippocampus (15). Below, we will describe specific features of these two neurogenic regions in rodents, since these are the mammals in which most studies have been reported.

### Neural Stem Cells of the Adult Subventricular Zone

**Figure 1:**
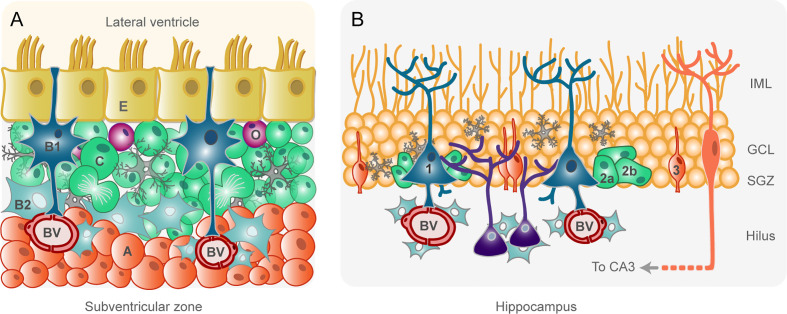
Schematic drawings of the adult rodent subventricular zone **(A)** and hippocampus **(B)**. Equivalent cell types within each niche are represented in the same color. **(A)**. Ependymal cells (type E, in yellow) separate the cerebrospinal fluid (CSF) in the lateral ventricle from the brain parenchyma. Type B1 cells (in dark blue) are neural stem cells with a basal process in contact with blood vessels (BV) and an apical process in contact with the CSF. B1 cells generate type C cells (in green) by asymmetric divisions. Type C cells are transit-amplifying intermediate progenitors that divide rapidly and produce neuroblasts (type A, in orange). Neuroblasts migrate in chains ensheathed by astrocytes toward the olfactory bulb, where they differentiate into mature interneurons. Type C cells can also produce oligodendrocyte progenitor cells (O, in purple). Other glial cells, such as astrocytes (type B2, in sky blue), and microglia (in gray) intervene in the control of subventricular zone neurogenesis. **(B)**. Neural stem cells (type 1, dark blue), located in the subgranular zone (SGZ) of the dentate gyrus of the hippocampus, harbor a basal process that contact BVs and numerous branches in the inner molecular layer (IML). Type 1 cells generate transit-amplifying non-radial type 2 cells (in green), which can be subdivided in type 2a and type 2b cells. Type 2 cells then give rise to a neuron-committed intermediate progenitor (type 3 cell, in orange). Type 3 cells generate fully functional granule cells in the granule cell layer (GCL) which, after maturation, develop dendritic arborization in the IML and axonal projection to the CA3. Microglia (gray) and astrocytes (sky blue) exert different roles in the control of neurogenesis.

NSCs of the adult rodent SVZ have the ability to generate neurons, astrocytes, and oligodendrocyte progenitor cells (OPCs) depending on niche signals (16). They are known as type B1 cells and the cell body is located under the layer of ependymal cells (type E cells) lining the ventricle (**Figure 1A**). B1 cells are polarized cells with a basal process contacting blood vessels and a non-motile primary cilium that contacts the cerebrospinal fluid (CSF) of the lateral ventricle (17, 18). Approximately 20% of B1 cells self-renew through symmetric divisions whereas ~80% generate transit-amplifying neural progenitors called type C cells (19) (**Figure 1A**). Type C cells are located deeper within the niche, close to the vascular network, are highly proliferative, and divide symmetrically before becoming neuroblasts (type A cells) (20) (**Figure 1A**). Type A cells are highly migratory and organize into chains to leave the SVZ through the rostral migratory stream to finally reach the olfactory bulb. Once in the olfactory bulb, type A cells change their migration pattern from tangential to radial through the cellular layers of the olfactory bulb to get to their target layer, where they ultimately differentiate into mature interneurons (14, 18, 21). In addition, a small proportion of type B1 cells can generate type C cells that express the oligodendroglial lineage marker Olig2 and give rise to OPCs (**Figure 1A**) that migrate to the corpus callosum and white matter tracts in the striatum and fimbria fornix (22, 23). SVZ-derived neuroblasts and OPCs can also migrate toward sites of brain injury, where they contribute to neural regeneration (24–26).

The SVZ also contains a population of proliferative astrocytes named type B2 cells (**Figure 1A**) that are located further beneath the ventricle but do not contact it. B2 cells isolate neuroblast chains from other cell types and help to shape the niche (20). Microglia are also an integral part of the SVZ niche (**Figure 1A**). Interestingly, SVZ microglia are clearly distinguished from microglia in other brain regions both antigenically and morphologically, and intervene in the control of neurogenesis through gap junctional communication and the release of soluble factors and extracellular vesicles [reviewed in (27)].

Additional constituents of the SVZ niche with relevance in the regulation of neurogenesis are endothelial cells. They have been reported to intervene in neuroblast migration and maturation (28) and to secrete soluble factors that regulate NSCs behavior (29). Moreover, direct cell-cell contact between B1 cells and endothelial cells is involved in the maintenance of B1 cells in a quiescent state (30).

### Neural Stem Cells of the Subgranular Zone of the Dentate Gyrus of the Hippocampus

The SGZ of the adult rodent hippocampus contains NSCs in different states of proliferation or differentiation, as well as other cell types that contribute to neurogenic functions. NSCs in the SGZ are radial glia-like cells called type 1 cells (**Figure 1B**) that are highly polarized, like radial glia and B1 cells (31). Type 1 cells harbor a primary cilium that contacts blood vessels in the hilus, a long process that extends through the granule cell layer and numerous branches in the inner molecular layer (**Figure 1B**). All these processes and branches allow type 1 cells to detect local neural activity from granule cells and from synaptic terminals, as well as signals from glial cells and blood vessels (31–33). Indeed, as it happens in the SVZ, the close proximity of blood vessels within the SGZ niche provides an abundant source of extrinsic factors that regulate proliferation, neuronal differentiation and survival (34). Unlike B1 cells, type 1 cells of the SGZ do not contact the CSF (31).

Under physiological conditions, type 1 cells are multipotent cells with low rate of division that can remain for long periods out of the cell cycle in a quiescent state (35, 36). NSC activation may lead to the expansion of the stem cell pool through symmetric and asymmetric self-renewal (35). The asymmetric divisions of hippocampal NSCs generate non-radial transit-amplifying cells (type 2 cells) destined to become neurons (37). There are two subtypes of type 2 cells, a glial-like type (type 2a) and a neuronally determined type (type 2b), which are negative and positive respectively for the immature neuron marker doublecortin (**Figure 1B**). Type 2 cells are highly proliferative and comprise the transition from a glia-like precursor cell to a neuronal determination, since they give rise to more committed intermediate progenitors (type 3 cells) that are constrained to a neuronal fate (15, 35, 38). Type 3 cells are neuroblasts with little proliferative activity, which migrate radially to the granule cell layer to generate fully functional granule cells (15) (**Figure 1B**). During the maturation stage, the newborn neurons extend their dendrites into the inner molecular layer and their axon to CA3 (15) (**Figure 1B**). Type 1 cells can also give rise to astrocytes through differentiation (37) but, unlike SVZ B1 cells, SGZ type 1 cells do not have the capacity to generate OPCs under normal conditions (39).

In the transition between type 2 and type 3 cells, there is a drastic decrease in newborn neurons, mediated by apoptosis. Apoptotic cells are rapidly cleared out through phagocytosis by microglia present in the adult SGZ niche (40) (**Figure 1B**). Astrocytes are also in close contact with different components of the SGZ niche and intervene in the regulation of the neurogenic process (41, 42).

As a brief summary, in rodents, neurogenic niches for hippocampal and subventricular zone neurogenesis exhibit many similarities but also clear differences. For instance, the hippocampus lacks ependymal cells and the whole process of neurogenesis is physically localized in the dentate gyrus (15, 31). In **Table 1**, we have summarized the main differences in the progenitor cell population between these two niches.

**Table 1:** Main differences between progenitor cells of the adult subventricular zone and the adult hippocampus in rodents.

	Rodent subventricular zone	Rodent hippocampus
**Neural Stem Cells**	B1 cells. Apical process in direct contact with cerebrospinal fluid (17, 18, 20)	Type 1 cells. Do not contact the cerebrospinal fluid (31)
**Intermediate Progenitors**	Type C cells. Give rise to oligodendrocyte progenitor cells and neuronal progenitors (18, 20, 23, 24)	Type 2 cells. Give rise to neuronal progenitors (15, 38)
**Neuronal Progenitors**	Type A cells. Migrate tangentially to the olfactory bulb to generate interneurons (14, 18, 21)	Type 3 cells. Migrate radially in the hippocampus to generate granule cells (15)

## Distinctive Features of the Human Subventricular Zone and the Human Hippocampus

**Figure 2:**
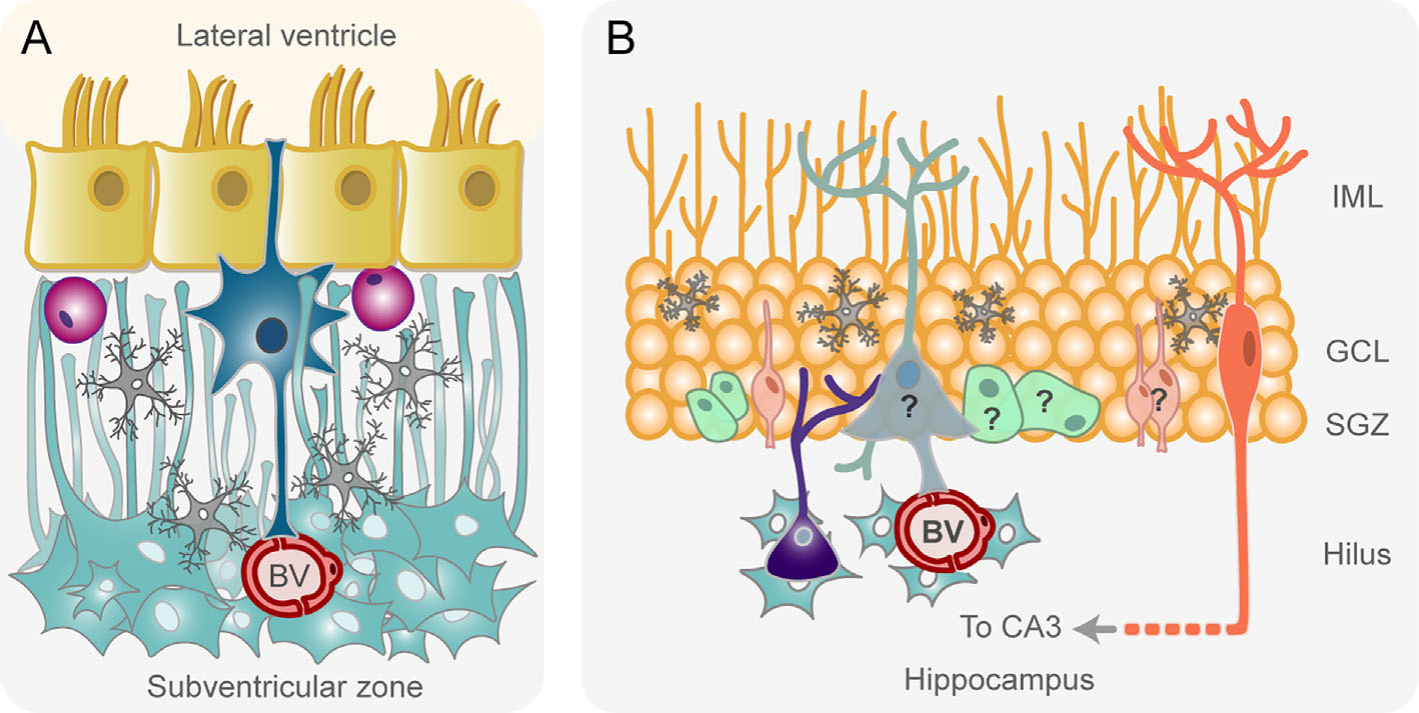
Schematic drawing of the adult human subventricular zone **(A)** and hippocampus **(B)**. **(A)** Astrocyte-like neural stem cells (NSCs, in dark blue) are located beneath the ependymal cell layer (in yellow) lining the lateral ventricles, within a hypocellular layer devoid of neuroblasts and transit-amplifying progenitor cells. Microglial cells in this layer are represented in gray. Putative oligodendrocyte progenitor cells generated from NSCs are shown in purple. NSCs contact the cerebrospinal fluid of the lateral ventricle and blood vessels (BV) of an adjacent layer consisted of a dense ribbon of astrocytes (pale blue) with processes in the hypocellular layer. **(B)** According to some studies (45, 46) the subgranular zone of the dentate gyrus of the adult human hippocampus contains radial glia-like neural stem cells (in pale blue) that generate proliferating intermediate neural progenitors (in pale green). These intermediate progenitors form neuronal committed progenitors (in pale orange) that become mature granule neurons (in orange). In contrast, other studies (47, 48) have reported the total absence of neuronal progenitors and immature neurons in the adult human subgranular zone. A question mark has been texted on these cells to symbolize this controversy. Other cell types of the niche are microglia (in gray) and astrocytes (in sky blue). GCL, granule cell layer; IML, inner molecular layer; SGZ, subgranular zone.

The SVZ of the adult human brain presents some peculiarities with respect to the SVZ organization of rodents described before. The cytoarchitecture significantly differs since, instead of a layer of abundant type C and type A cells, characteristic of the rodent SVZ, an almost acellular layer devoid of neuroblasts is located beneath the ependymal cells in the human SVZ (43, 44) (**Figure 2A**). Adjacent to this hypocellular layer there is a dense ribbon of astrocytes that extend processes across the hypocellular gap layer to maintain contact with the surface of the lateral ventricle (**Figure 2A**). Astrocyte-like NSCs are located in the hypocellular gap layer (43) (**Figure 2A**). This appearance is adopted as early as 18 months of age, when both proliferative activity and the expression of markers of immature neurons are largely depleted (49). In addition, during this limited postnatal period of neurogenesis, not all neuroblasts generated in the human SVZ are destined to the olfactory bulb, since many of them migrate tangentially to the prefrontal cortex (49). The incorporation of newborn neurons in the human olfactory bulb is nearly extinct by adulthood (50, 51), which may be related to the reduction in the dependence of olfaction manifested in humans (50). Surprisingly, the scarce neuroblasts formed in the adult human SVZ migrate to the striatum, where they differentiate into interneurons, a phenomenon that has been observed mainly in response to cell loss due to injury (49, 50, 52). Importantly, newly generated cells from NSCs in adult human SVZ are mainly oligodendrocytes, not neurons (50, 52), which suggests that the oligodendrogenic process and its corresponding myelin maintenance acquires more relevance in the human brain when compared to other mammalian brains (**Figure 2A**).

With respect to the human hippocampus, proliferating neural progenitor cells and newly generated neurons were described in the adult human dentate gyrus by the end of the last century (45) **(** **Figure 2B** **)** and their existence has been suggested, even in the aged brain, in recent studies (46, 53, 54). The preservation of neurogenesis throughout life in healthy older people seems to be important to maintain cognitive function (46, 54). Remarkably, a larger proportion of hippocampal neurons are subject to exchange in humans in comparison to the mouse (55). However, other studies have argued against the existence of adult neurogenesis in humans (47, 48) **(** **Figure 2B** **)**. Sorrells and colleagues showed that proliferating progenitors and young neurons in the dentate gyrus declines sharply during the first year of life and only a few isolated young neurons can be observed by 7 and 13 years of age (47). Therefore, an interesting debate has been established about the existence or not of neurogenesis in the adult human hippocampus. The contradictory hypothesis seems to be due to differences in the treatment of human postmortem tissue and in the neuronal markers used in diverse studies (56). Thus, while some authors used doublecortin as a marker for young neurons (54), others affirm that this marker is not specific to newborn neurons, since they continue to express it as they differentiate, and it is also expressed by non-neuronal glial cells (47). Therefore, there is a clear need to unify criteria in order to have a better understanding of the neurogenic process in the hippocampus of the adult human brain.

## Different Theories on the Cell of Origin of Glioblastomas

**Figure 3:**
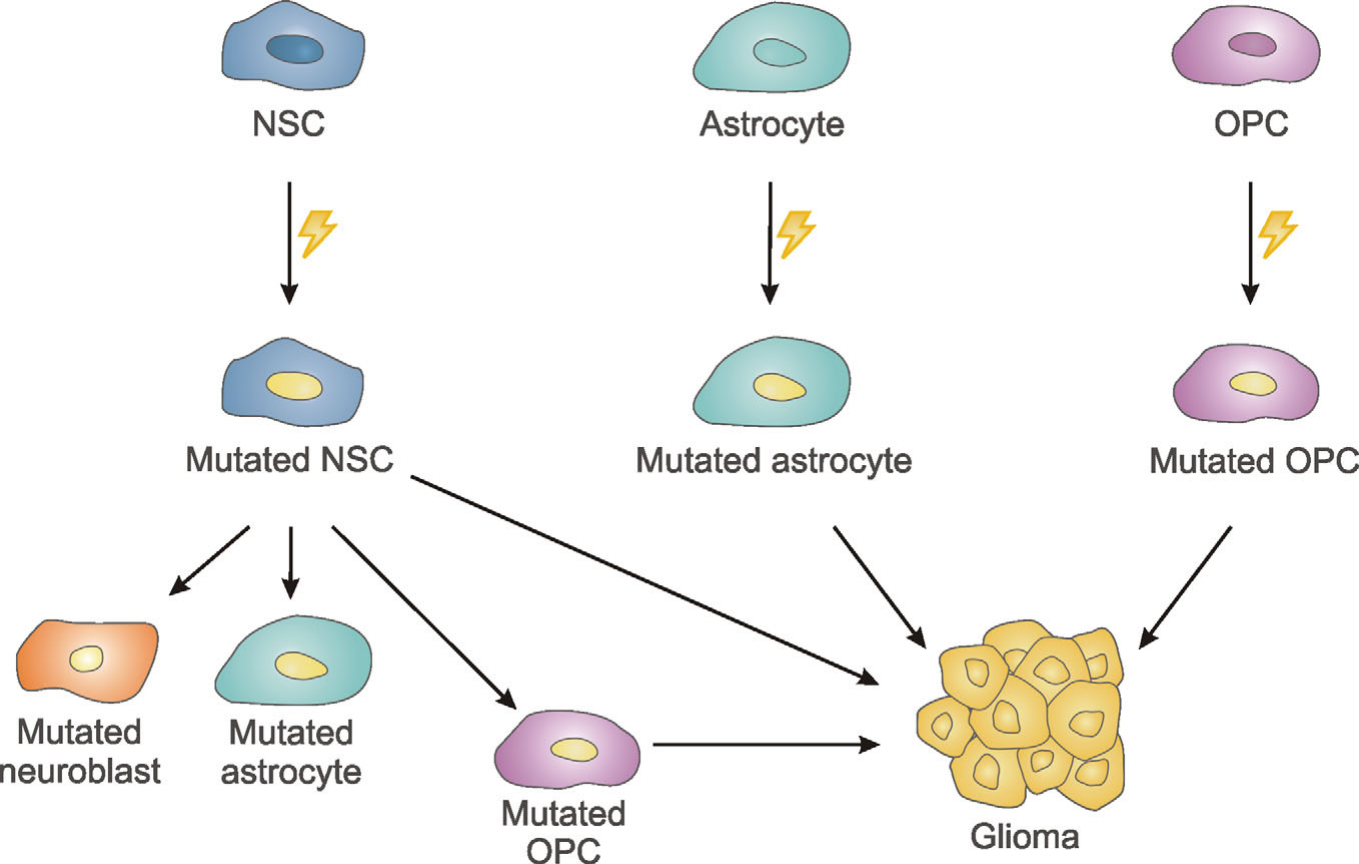
Schematic drawing summarizing different theories on the cell of origin of glioblastomas. Neural stem cells (NSC) of the subventricular zone, astrocytes and oligodendrocyte precursor cells (OPCs) of the brain parenchyma might acquire cancer-driver mutations leading to gliomas. Additionally, NSC-derived mutations can be transmitted to their progeny generating mutated neuroblasts and mutated astrocytes, that do not likely give rise to tumor formation, or mutated OPCs with the ability to grow aberrantly.

The cell type that originates this devastating tumor has been a subject of debate in the last few years, with data supporting NSCs, astrocytes or OPCs as putative candidates (57). For instance, several reports in mice have demonstrated that the knockout/knockdown of tumor suppressor genes (e.g. *PTEN*, *P53*, *NF1*, *retinoblastoma protein RB1*) in NSCs from the SVZ leads to glioma formation (58, 59) **(** **Figure 3** **)**. Genetic modifications in astrocytes or in NSC-derived astrocytes leading to combined inactivation of several tumor suppressor genes or in driver oncogenes (*EGFR)* are also capable of initiating gliomagenesis in mice (60–62) **(** **Figure 3** **)**, although other authors have reported that oncogenic mutations in mature astrocytes do not contribute to the formation of gliomas (58, 59). OPCs have also been considered as the cell population that originates this deadly cancer. Thus, inactivation of *p53* and *NF1* (63) *or p53, NF1* and *PTEN* (64) in adult OPCs induce glioma formation in mice **(** **Figure 3** **)**.

Latest data obtained from human patients have reinforced the candidacy of NSCs from the SVZ, as the possible cell type carrying the cancer-driving mutations of GBM (11). Lee et al. (11) have convincingly demonstrated that tissue from the tumor-free SVZ of IDH-wild type GBM patients contained low-level GBM driver mutations (in *TP53, PTEN* or *EGFR*), that were found in the dominant clones of its matching tumors (11). In addition, *TERT* promoter mutations were identified in all the patients with GBM that had driver mutations in tumor-free SVZ tissue. In the same publication, authors performed experiments in mice to support their findings in human tissue. Thus, they generated a mouse model with *p53*, *PTEN* and *EGFR* mutations in NSCs from the SVZ and showed that mutant cells migrated from the SVZ to distant regions of the brain and eventually developed high-grade glioma. Interestingly, mutated cells that migrated toward the olfactory bulb differentiated into mature neurons and did not lead to gliomas. The aberrant growth of the mutated OPC lineage, but not of the mutated astrocytic lineage, was found to be involved in the glioma formation (11). Previous experiments performed in a mouse model of glioma led to similar conclusions (65). In their article, Liu *et al.* induced *p53/NF1* mutations in NSCs to model gliomagenesis in mice and analyzed mutant NSCs and their progeny at premalignant stages (65). They demonstrated aberrant growth of OPCs, but not of NSCs or any other NSC-derived lineages as neurons or astrocytes. Upon tumor formation, it was confirmed the OPC nature of the tumor cells. Consistently, gliomas were also formed when these mutations were performed directly in OPCs. These results indicate that while both SVZ NSCs and OPCs could be the cell of origin of GBM, OPCs could rather constitute the population involved in the generation of the tumor mass, this is, the “tumor-propagating cell population”.

Further evidence supporting SVZ NSCs as cell of origin of GBM comes from striking similarities in marker expression between NSCs and GBM cells. Some typical NSC markers identified in GBMs are: nestin, glial fibrillary acidic protein (GFAP), Sox2, CD44, and CD133 (prominin-1) (66–68). Nonetheless, characteristic expression profiles of OPCs such as neuronal glial antigen 2 (NG2), Olig2 and platelet-derived growth factor receptor α (PDGRα) can also be found in some GBM subtypes (69–71). This indicates that NSCs and OPCs may give rise to distinct GBM subtypes contributing to the intertumor heterogeneity of GBMs (64).

Remarkably, in spite of sharing many features with SVZ NSCs, NSCs residing in the hippocampus have barely been involved in glioma formation. Friedmann-Morvinski and colleagues showed that transduction of hippocampal NSCs with oncogenic lentiviral vectors gave rise to malignant glioma in mice (72) but, surprisingly, no further data have been published supporting a role for hippocampal NSCs in glioma formation. In addition, the hippocampus is a region spared from GBM (12). Mughal *et al.* analyzed the invasion patterns of glioma cells in two mouse models of invasive GBM and in magnetic resonance images from GBM patients, and showed that, in all cases, despite extensive tumor cell infiltration in hippocampal adjacent structures, very few tumor cells were observed within the hippocampus itself (12). The absence of NSCs in the adult human hippocampus reported by some authors (47, 48) may account for the lack of hippocampal involvement in glioma formation or invasion. But if adult human hippocampal NSCs do exist, as other authors have shown (45, 46, 53, 54), alternative explanations should be provided for the scarce literature relating hippocampal NSCs with glioma development.

## Possible Reasons Underlying the Lack of Data Relating Hippocampal Neural Stem Cells With Gliomagenesis

### Differences in NSC Fate in Human SVZ and Hippocampus: OPCs Are More Likely to Develop Gliomas Than Neuroblasts

OPCs, also referred to as NG2 cells, represent a major resident glial cell population in the mammalian CNS with the ability to generate myelinating and non-myelinating oligodendrocytes [reviewed in (73)]. Most OPCs are generated during development from the ventral germinal zones of medial and lateral ganglionic eminences (74). During both the postnatal period and the adulthood, additional OPCs are generated from NSCs of the SVZ (16, 22). OPCs constitute the major dividing cell population of the adult mouse and human brain (70, 75, 76). Moreover, in rodents, the proportion of NG2 cells that is actively cycling (~50%) does not decrease with age, although the cell cycle time does (less than 2 days at postnatal day 6 (P6), ~9 days at P60, and ~70 days at P240) (77). Proliferation of adult OPCs and subsequent differentiation into myelinating oligodendrocytes can be activated by stimuli such as neuronal activity or brain injury (76, 78, 79). As mentioned earlier, adult OPCs can be reactivated to a highly proliferative state by oncogenic mutations and give rise to malignant gliomas (63, 64). Alternatively, OPCs generated from SVZ NPCs carrying cancer-driver mutations can also proliferate aberrantly to generate GBM (11). Therefore, adult OPCs are cells to take into account in gliomagenesis, either for being the cell that acquires the initial oncogenic mutations or for being the cell type that propagates the tumor (**Figure 3**).

As mentioned earlier, adult human SVZ NSCs can form neuroblasts that migrate to the striatum (52), but their main progeny are OPCs (50, 52). In contrast, NSCs of the human hippocampus exclusively generate neuronal progenitors (46, 53, 54). This suggests that, if a cancer-driver mutation occurs in adult human NSCs of either origin, the mutation would be transmitted to their progeny, neuroblasts and OPCs in the SVZ, but only neuroblasts in the hippocampus. In mice, mutated OPCs can act as tumor-propagating cells (11, 65) whereas neuroblasts derived from mutated SVZ NSCs migrate to the olfactory bulb and do not develop gliomas (11). It is reasonable to think that neuroblasts born from putative mutated NSCs of the adult human SVZ would not grow aberrantly either after migration to the striatum. In support of this notion, Alcantara Llaguno *et al.* (80) assessed the tumor-initiating potential of late-stage neuronal progenitors, neuroblasts and differentiated neurons, in which the tumor suppressors genes *Nf1*, *p53*, and *Pten* were inactivated by Cre recombinase-mediated gene targeting. They showed that the susceptibility of malignant transformation decreases as the lineage restriction increases. Although cellular and molecular defects were detected as a consequence of the inactivation of the tumor suppressor genes, no evidence of glioma formation was observed in any case (80).

To sum up, if we assume that the population harboring the initial cell mutations is the same that the population that develops the tumor, then, NSCs from both SVZ and hippocampal origin might have similar chances to develop gliomas. But if the tumor-propagating cell population differs from the cell of origin, then it is more likely that OPCs originated from mutated SVZ NSCs, and not neuroblasts born from mutated hippocampal NSCs, proliferate aberrantly to form a tumor mass (**Table 2**).

**Table 2:** Analysis of possible factors determining why neural stem cells of the adult human subventricular zone might be more susceptible to malignant transformation than neural stem cells of the adult human hippocampus.

Human subventricular zone	Human hippocampus
NSCs mainly produce OPCs (50, 52)	NSCs mainly produce neuroblasts (45–46)
Niche factors favor neuroblast migration (18, 21)	Niche factors favor neuroblast differentiation and neuronal maturation (15, 18)
Chemoattractive for glioma cells (81)	Chemorepellent for glioma cells (12)
Microglia are supportive for neurogenesis (82, 83)	Microglia phagocyte apoptotic neuroblasts (40)
NSCs contact the CSF (17, 18, 84, 85)	NSCs do not contact the CSF (31)
NSCs do not express the tumor suppressor gene *HOPX* (86)	NSCs express *HOPX* (86)
Mutations in *TERT* might increase senescence (11, 87)	Mutations in TERT might not be relevant to senescence (88)

CSF, cerebrospinal fluid; HOPX, homeodomain-only protein; NSCs, neural stem cells; OPCs, oligodendrocyte precursor cells; TERT, telomerase reverse transcriptase.

### Neuroblast Final Differentiation Takes Place in the Hippocampus, but Not in the SVZ

As explained in detail in a former section, one of the most remarkable differences between the SVZ and the hippocampus neurogenic niches is that in the SVZ, neuroblasts generated from transit amplifying progenitors do not differentiate into neurons within this region. Instead, they migrate tangentially toward the olfactory bulb where their final differentiation occurs (14, 18, 21). In contrast, neuroblasts generated from intermediate progenitors in the hippocampus differentiate into mature granule cells within the same niche (15, 18). There is a high variety of factors that control the important step of final differentiation, ranging from intrinsic factors (specific to the progenitor cell population) to extrinsic factors (as the result of the surrounding microenvironment) (18). This cocktail of elements together with their induced molecular responses will determine whether a neuroblast initiates tangential migration toward the olfactory bulb (as it happens in the SVZ) or differentiates (as it happens in the hippocampus). The existence of factors promoting neuronal differentiation in the hippocampus might constitute another possible explanation for the lack of literature reporting tumorigenicity of hippocampal NSCs (**Table 2**). In support of this notion, two recent studies have demonstrated that reprogramming GBM cells into neurons suppresses tumor growth and prolongs survival in mice implanted with human GBM cell lines (89) or with patient-derived GBMs (90). Strikingly, the reprogramming of GBMs into neurons achieved by treatment with mTOR (mammalian target of rapamycin) and ROCK (Rho-associated kinase) inhibitors prevented GBM local recurrence (89). These are important findings in terms of therapeutics development for GBM patients.

### The Hippocampal Niche Does Not Favor GBM Invasion

Tumoral cells both in GBM animal models and in patients, follow preferred migratory paths (e.g. optic and pontine white matter structures) and avoid the hippocampus despite being closely located to the tumor (12). However, the SVZ is revealed as one of the most tumor-infiltrated regions in the same study. Other reports have demonstrated that the SVZ is a region of preferred migration for both IDH1-wild type and IDH1-mutant GBMs (91). The presence of molecular cues derived from the specific composition of the extracellular matrix of these regions might guide tumor cell migration toward or away from them (**Table 2**). It is reasonable to think that factors released by the SVZ neurogenic niche might be chemoattractive for glioma cells. Indeed, Qin *et al.* have shown that NSCs of the SVZ secrete pleiotrophin, which forms a chemoattractant complex with other proteins that promote glioma invasion (81). Interestingly, these authors described that pleiotrophin is undetectable in the hippocampus. Therefore, pleiotrophin might be an important molecule involved in the preferred migration of GBM cells toward the SVZ. Another SVZ-released molecule implicated in the stimulation of glioma invasion is CXCL12 (C-X-C motif chemokine 12) (92). In addition, in the SVZ, CXCL12 can mediate GBM resistance to radiation therapy (93). However, this chemokine is also expressed in the hippocampus (94), where it intervenes in the support of newborn neuron maturation (95). Thus, it is less likely that this chemokine might act as a preferred cue for glioma cell migration. Besides, in adult human SVZ, the layer where NSCs reside (hypocellular gap layer) is almost an acellular layer filled with astrocytic processes that provides and environment with fewer hindrances to invasion.

Therefore, the difference for preferred migration exists, but is it connected to a higher lethality of the tumor? In a retrospective study with a cohort of 207 adult patients who underwent cytoreductive surgery for GBM followed by chemotherapy and/or radiation, the authors showed that GBMs contacting the SVZ show earlier recurrence and lower survival than those contacting the SGZ of the hippocampus (96). This work concluded that the SVZ has unique properties that contribute to GBM pathobiology.

On the other hand, the fact that the hippocampus is a region spared from GBM invasion indicates that molecular cues within this niche are not favorable either for tumor chemoattraction or for tumor support (**Table 2**). Identification of signals from the hippocampus that do not support tumor formation as well as the chemoattractants from the SVZ might be of great importance for the development of novel therapeutic strategies for GBM.

### Microglia in the Hippocampus Do Not Provide Trophic Support to NSCs

Microglial cells are a main constituent of the adult neurogenic niches (**Figure 1**) although the role exerted by these immune cells differs between the SVZ and the hippocampus. Thus, microglia in the postnatal and adult SVZ provide trophic support for newly-generated neuroblasts and promote their migration toward the olfactory bulb (82, 83), whereas microglia in the hippocampus are involved in the control of neurogenesis through phagocytosis of newborn cells that become apoptotic (40). This process of phagocytosis of apoptotic cells is mediated by purinergic “find me, eat me” signals (97). Interestingly, microglia in the SVZ and in the rostral migratory stream show very low level of purinergic receptors, which allows them to avoid inappropriate activation in response to locally active purines that might result in undesired phagocytosis of neuroblasts before they reach the olfactory bulb (83). Therefore, microglia have a “classical” immune function in the hippocampus whereas in the SVZ acquire a different phenotype to support neurogenesis. It is important to highlight that microglia/macrophages of the GBM microenvironment adopt a tumor-supportive phenotype characterized by the release of anti-inflammatory molecules, trophic factors and metalloproteinases [reviewed in (98)]. It is therefore reasonable to think that, if a driver mutation occurs in SVZ NSCs, adjacent microglia might act as supporter cells favoring the proliferation and migration of these cells. However, if this were the case in the hippocampus, niche microglia would not have a permissive phenotype for tumor evolution (**Table 2**).

### NSCs of the Hippocampus Do Not Contact the CSF

As mentioned before, NSCs in the SVZ are in direct contact with the CSF through a small apical process harboring a primary cilium (**Figures 1A** and **2A**) that is likely involved in signal transduction (84, 85). In contrast, in the SGZ of the hippocampus, type 1 cells do not contact the CSF (31). This noticeable difference between the two adult neurogenic niches might determine a higher susceptibility of NSCs of the SVZ to malignant transformation or uncontrolled proliferation (**Table 2**).

Soluble factors in the CSF (which are released by epithelial cells of the choroid plexus) are important not only for the maintenance of NSC quiescence in the SVZ, but also for the regulation of multiple aspects of the adult NSC behavior and their progeny (84, 99–101). Recently, de Sonnaville et al. have shown that human ventricular CSF increases proliferation of SVZ NSCs (102). Some of the soluble molecules contained in the CSF are growth factors which have been reported to be involved in the stimulation of NSC proliferation, such as insulin-like growth factor 2 (IGF-2) (103), transforming growth factor-β (TGF-β) (104), vascular endothelial growth factor A (VEGFA) (105), leukemia inhibitory factor (LIF) (106) or endogenous ligands of the EGFR (107). Noteworthy, these factors have also been shown to contribute to glioblastoma growth or support (103, 108–111). Consequently, the direct contact of SVZ NSCs with these mitogens might confer them growth advantages if the concentrations of these factors were increased or if NSCs had acquired somatic mutations which made them more responsive to these proliferative signals.

Lately, other constituents of the CSF have gained much importance in intercellular communication: the extracellular vesicles (EVs). EVs are small membrane vesicles (30 nm–10 μm) secreted by almost all cell types that are implicated in the transfer of mRNAs, microRNAs, proteins and lipids between cells and thus are able to modify the function of recipient cells. Isolation of membrane vesicle-enriched fractions and further proteomic studies have demonstrated the presence of EVs in the human CSF (112, 113). This might be relevant since EVs can transfer oncogenic cargo to recipient cells. For instance, EGFRvIII contained in EVs released by glioma cells has been shown to be transferred to indolent glioma cells in which they induce oncogenic activity (114). Similarly, Gutkin *et al.* reported EV-mediated horizontal transfer of hTERT mRNA from cancer to non-cancer cells (115). These observations raise the hypothesis that NSCs may be susceptible to malignant conversion *via* EV-mediated molecular transfer.

Therefore, NSCs in the SVZ, due to their direct contact with the CSF, are exposed to growth factors and EVs that might increase their susceptibility to aberrant growth or malignant transformation (**Table 2** **).**

### Adult Hippocampal, but Not Adult SVZ NSCs, Express the Tumor Suppressor Gene *HOPX*.

SVZ and hippocampal NSCs share astroglial features and expression of numerous molecular markers (18). However, there are some differences that might be relevant in terms of susceptibility to malignant transformation. One of these differentially-expressed proteins is HopX (homeodomain-only protein), an atypical homeodomain protein that cannot bind DNA and exerts its actions by interacting with serum responsive factor (SRF) and blocking its transcriptional activity (116). *HOPX* gene is selectively expressed by quiescent NSCs of the adult hippocampus, but not by adult SVZ NSCs (86). Specifically in the dorsal SVZ, this protein is present during embryonic and postnatal stages in NSCs primed toward astrocytic fates but declines to nearly undetectable levels in adulthood (117). In the adult hippocampus, HopX has been described to intervene in the regulation of neurogenesis by promoting apoptosis of NSCs. Remarkably, *HOPX* expression is down-regulated in GBMs and on the other hand, a cell-permeable version of HopX protein with gain of function characteristics causes an increase in apoptosis in a subset of GBM cells and a decrease in clonogenicity (118). It is worth mentioning that *HOPX* expression is lost or down-regulated in other cancers as well (119, 120). Hence, we hypothesize that the restricted expression of HOPX in adult NSCs of the hippocampus might confer tumor-suppressive properties to this population of cells (**Table 2**).

### Mutations in the Catalytic Subunit of Telomerase Reverse Transcriptase (TERT) Might Have Different Implications in SVZ NSCs Than in Hippocampal NSCs

Telomere length is essential for the prolonged persistence of stem cell functions in organs with extensive cell turnover (121, 122). The maintenance of telomere length is mediated by telomerase, an enzyme that adds nucleotides to the end of the chromosomes and prevents the replication-dependent loss of telomere and cellular senescence (122, 123). The active telomerase enzyme consists of a protein component TERT that serves as catalytic subunit, and a telomerase RNA component (TERC) (124, 125). Human somatic cells lack telomerase activity, which can be considered as a tumor suppressor mechanism since it prevents unlimited clonal expanding. Accordingly, 90% of human tumors are telomerase positive (126).

In the context of GBM, Lee and colleagues demonstrated that all the IDH-wild type GBM patients with driver mutations in tumor-free SVZ tissue also presented mutations in the *TERT* promoter in this tissue (11). Indeed, they suggested that mutation-driven activation of *TERT* in SVZ NSCs might be the earliest event by which these cells, having limited self-renewal capability, are able to avoid replicative senescence thereby increasing their chances of acquiring other driver mutations over time (11). Hippocampal NSCs might also acquire mutations in *TERT* that could increase their senescence and the subsequent possibility of becoming tumorigenic. However, current data do not support this hypothesis, as will be discussed below.

First, in humans, *TERT* mutations have been reported to occur in NSCs of normal or non-cancer aged hippocampus at much lower rates than those found in SVZ NSCs of IDH-wild type GBM (11). Second, overexpression of TERT in mouse hippocampal NSCs does not lead to tumor formation (88). Third, in rodents, the SVZ and olfactory bulb have significantly higher levels of telomerase activity than the hippocampus (87). And fourth, in the mouse hippocampus, TERT exerts additional roles independent of its telomerase activity (88). Thereby, by knockdown and overexpression of TERT, Zhou and colleagues (88) demonstrated that TERT is required for neural circuit integration of hippocampal newborn neurons, as well as for spatial memory processing. TERT actions through non-canonical pathways have not yet been described in SVZ NSCs.

These data suggest that putative mutations in *TERT* leading to increased telomerase activity would have more chances to induce glioma formation in SVZ NSCs than in hippocampal NSCs (**Table 2**).

## Conclusions

Experiments performed in mice have revealed that oncogenic mutations in NSCs of the SVZ, astrocytes or OPCs can all lead to glioma formation. However, recent data obtained from GBM patients have reinforced the hypothesis that NSCs of the SVZ are the cell of origin of IDH-wild type GBM. Since NSCs are anatomically restricted in the SVZ whereas astrocytes and OPCs are widely distributed in the brain, the clinical implications on the diagnosis and therapy for these lethal tumors may considerably vary depending on the cell of origin.

NSCs of the hippocampus, however, have not been associated either to the origin or to the propagation of GBM. Throughout this article we have provided some possible explanations for this fact that are summarized in **Table 2**.

One of the reasons supporting SVZ-derived NSCs as cell of origin of IDH-wild type GBM rely on the specific progeny of NSCs of every neurogenic niche. SVZ-derived NSCs mainly produce OPCs in the human brain, whereas hippocampal-derived NSCs produce neuroblasts. Progenitors committed to a neuronal fate are less prone to develop gliomas than those committed to the oligodendroglial lineage. In addition, factors within the SVZ niche might be more permissive for aberrant tumoral cell migration and growth than those present in the SGZ niche. Other possibilities such as differential expression of tumor suppressor genes and differential effects of *TERT* mutations and roles between SVZ and SGZ NSCs can be taken into account to find possible explanations for the higher susceptibility of malignant transformation of SVZ NSCs.

## Author Contributions

EM conceived and designed the manuscript. ÁF-L, SM, and EM wrote the manuscript. BB-T and MD-L designed and edited the figures. RM revised the manuscript. All authors contributed to the article and approved the submitted version.

## Funding

Funded by VI Plan Propio de Investigación (Universidad de Sevilla) Grant number 2020/0000081.

## Conflict of Interest

The authors declare that the research was conducted in the absence of any commercial or financial relationships that could be construed as a potential conflict of interest.
